# The radiographic soft tissue thickness is associated with wound complications after open reduction and internal fixation of patella fractures

**DOI:** 10.1186/s12891-022-05498-0

**Published:** 2022-06-06

**Authors:** Kai Song, Bowen Zhu, Qing Jiang, Jin Xiong, Hongfei Shi

**Affiliations:** 1 State Key Laboratory of Pharmaceutical Biotechnology, Division of Sports Medicine and Adult Reconstructive Surgery, Department of Orthopedic Surgery, Nanjing Drum Tower Hospital, The Affiliated Hospital of Nanjing University Medical School, 321 Zhongshan Road, Nanjing, 210008 Jiangsu People’s Republic of China; 2 Laboratory for Bone and Joint Disease, Model Animal Research Center (MARC), Nanjing University, Nanjing, 210093 Jiangsu People’s Republic of China; 3 Department of Orthopedic Surgery, Nanjing Drum Tower Hospital, The Affiliated Hospital of Nanjing University Medical School, 321 Zhongshan Road, Nanjing, 210008 Jiangsu People’s Republic of China

**Keywords:** Patella fracture, Open reduction and internal fixation, Wound complication, Soft tissue

## Abstract

**Background:**

Displaced patella fracture is commonly treated with open reduction and internal fixation (ORIF). Wound complications after surgery often lead to prolonged hospitalization and dissatisfaction of patients. Periarticular adiposity and swelling may be associated with wound complications. The purpose of this study is to determine the relationship between periarticular soft tissue thickness and wound complications after ORIF of patella fractures.

**Methods:**

We retrospectively studied 237 patients undergoing ORIF for patella fractures from June 2017 to February 2021 at our institution. We established periarticular soft tissue thickness ratio (PSTTR) to evaluate soft tissue status on lateral knee X-ray radiographs. Univariate analysis was performed to identify the association between PSTTR and postoperative wound complication. A receiver-operating characteristic (ROC) curve analysis was performed to evaluate the predictive value of PSTTR.

**Results:**

The incidence of postoperative wound complication was 10.5%. Minor wound complication occurred in 24 patients, and major wound complication occurred in one patient. The average femoral PSTTR (fPSTTR) was 0.94 ± 0.17 and the average tibial PSTTR (tPSTTR) was 0.66 ± 0.16. fPSTTR proved to be associated with postoperative wound complication. In the ROC analysis of fPSTTR predicting postoperative wound complication, the area under curve (AUC) was 0.676, which indicated a moderate predictive value.

**Conclusions:**

PSTTR was a feasible method to assess periarticular soft tissue. The increased fPSTTR was associated with wound complications after ORIF of patella fractures.

## Background

Patella is the largest sesamoid bone of the human body and an important component of the extensor mechanism of the knee. Patella transfers the tensile force from the quadriceps to the patellar tendon, which results in the knee extension. Patella fractures account for about 1% of all skeletal injuries [1]. The reported incidence of patella fracture ranges from 10.5–16.5/100,000/year [2–4]. Peak age of affected patients is 10–19 years old in males and 60–80 years old in females [2]. A direct blow is the most common cause of patella fractures. Besides, the patella can be damaged indirectly by a sudden tensile force with hyperflexion of the knee.

Open reduction and internal fixation (ORIF) is commonly used to treat displaced patellar fractures. The goals of surgery are to restore the congruency of patellofemoral joint and the function of extensor mechanism. However, symptomatic complaints and functional deficits may be caused by postoperative complications, which include loss of reduction or fixation, symptomatic hardware, nonunion, infection, and soft tissue problems [5–7]. The incidence of infection following patella fracture operation varies from 1.5–10%, mainly because of different surgical techniques [6–8]. Postoperative infections usually require local wound care, antibiotic therapy, operative debridement or hardware removal, which may prolong hospital stay, increase treatment expense, and decrease patient satisfaction. However, there are limited studies to explore the risk factors for wound complications in these patients.

Obesity has widely proven to be a risk factor for infection after orthopaedic surgeries [9], especially in the lower limb fracture surgeries, including acetabular fracture [10, 11], femoral neck fracture [12], distal femur fracture [13], tibial fracture [14], ankle fracture [15], and calcaneal fracture [16]. Therefore, we hypothesize that obesity may be associated with wound complication after patella fracture operation. Body mass index (BMI), which is a convenient tool to evaluate obesity in clinical practice, is widely used in the previous studies. However, BMI has limitations, including neglecting variations in body habitus and local adiposity. Besides, surrounding soft tissue of the knee may be swelling in the patients with patella fracture, which may also increase the risk of wound complication. However, there is a lack of an accessible and feasible tool to assess periarticular soft tissue envelope size. Almost all of the patients undergoing patella fracture operation receive imaging examination preoperatively, especially knee X-ray radiograph, which may serve as a potential tool.

In this study, we aimed to develop a radiographic measurement to evaluate the thickness of surrounding soft tissue of the knee and determine its relationship with wound complications after ORIF of patella fractures.

## Methods

This retrospective study was approved by the Institutional Review Board (IRB) of our institution. All consecutive patients undergoing ORIF with tension band technique for closed patella fracture during the period of June 2017 to February 2021 at our institution were included in this study. Exclusion criteria included nonstandard lateral knee X-ray radiograph, incomplete data collection, ipsilateral lower limb combination injuries, and identified preoperative infection. The initial capture population included 270 consecutive patients. Eighteen patients without an acceptable preoperative lateral knee X-ray radiograph were excluded. An acceptable radiograph was taken when posterior femoral condyles overlapped and the knee was flexed between 10° and 30°. Fifteen patients were excluded because of combination injuries or identified preoperative infection.

We reviewed the medical records of these patients and collected their demographic and clinical information, including gender, age, BMI, hypertension, diabetes, smoking status, AO/Orthopaedic Trauma Association (OTA) classification, time to operation, time to X-ray test, surgeons, and preoperative level of C-reactive protein (CRP). All surgeries were performed by four orthopedic trauma fellowship trained surgeons with greater than 10 years of experience. Under general anesthesia, the patient was placed in a supine position. A tourniquet cuff was applied and a midline longitudinal incision centered over the patella was conducted. After exposure of the fracture site, hematoma was cleared and the articular cavity was irrigated with normal saline. The fractured articular surface was carefully inspected to reduce the articular impaction. The proximal and distal fracture fragments were then reduced with two large towel clips. One or two parallel longitudinal Kirschner wires were placed to provide provisional fixation. After articular reduction was assured with intraoperative C-arm, one single-looped cable cerclage (Φ 1.7 mm, Synthes, Switzerland) was applied around the reduced patella using an epidural puncture needle as the cable passer. Then an anterior tension band in a vertically oriented figure of 8 configuration was applied using another cable. If there were retinacular tears, absorbable braided suture would be used to repair the torn retinaculum after the fracture was fixed. Electrocautery was used after tourniquet release to archive hemostasis. The wound was irrigated thoroughly and closed in layers. Figure of 8 nonabsorbable suture was used to close arthrotomy. Subcutaneous closure was conducted using 2–0 vicryl, and skin closure was conducted using staples. Subcutaneous drainage was not used routinely. Compression bandaging was applied to avoid hemarthrosis. All patients received routine prophylactic intravenous antibiotics (cefazolin sodium, 1 g twice daily, or clindamycin, 0.6 g twice daily) within 24 h after surgery. Patients were followed up by routine clinic visit or telephone contact to collect information about wound complications. The mean follow-up was 25.9 ± 13.5 months (range: 12–56 months). Wound complications were defined as a deviation of the postoperative wound evolution that required non-surgical or surgical intervention, including wound seroma, hematoma, dehiscence, and surgical site infection. Wound complications were further categorized as major or minor complications [17]. A minor complication referred to the requirement for any non-surgical intervention, such as intensive wound care and use of antibiotics. A major complication was defined as the need for operative treatment, such as debridement with or without hardware removal.

**Fig. 1:**
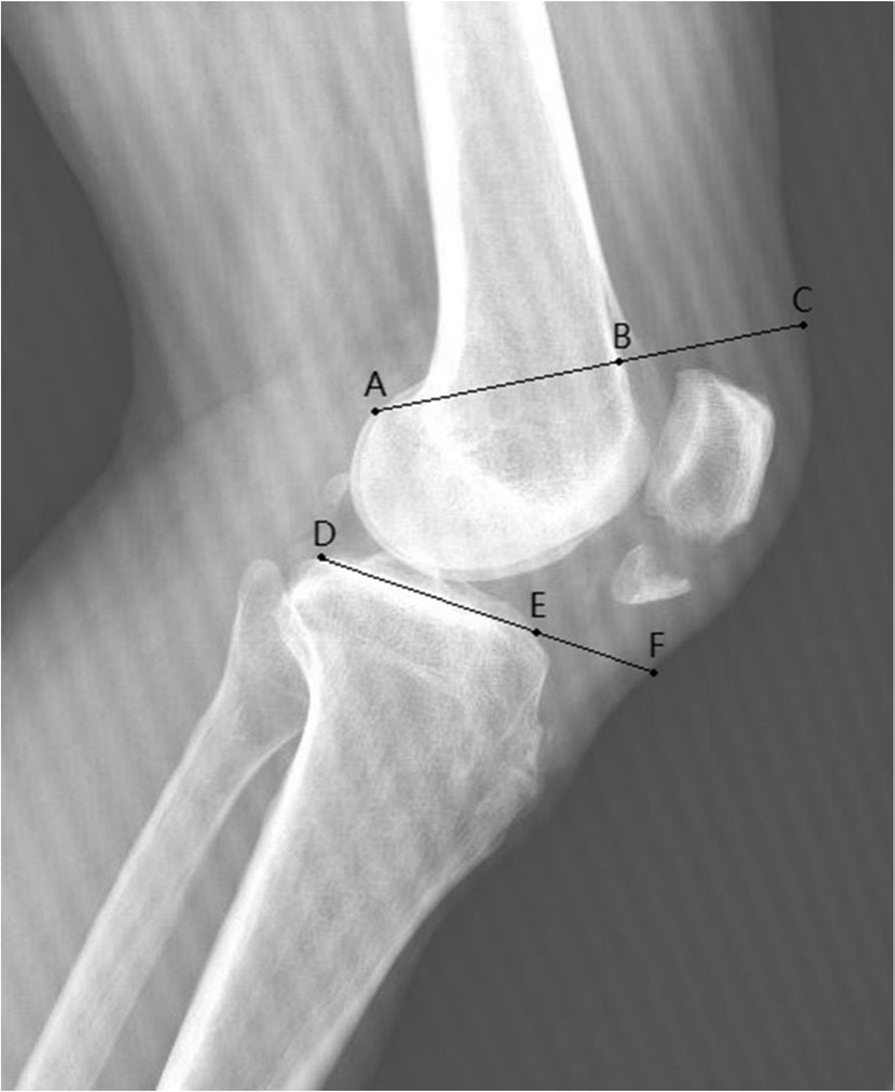
The measurement of PSTTR. This figure shows the method to measure and calculate PSTTR. fPSTTR is BC divided by AB, and tPSTTR is EF divided by DE

Preoperative lateral knee X-ray radiographs were viewed and measured using Phillips iSite PACS system. Several studies have used different methods to evaluate periarticular soft tissue thickness and explored its association with wound complications after total knee arthroplasty [17–19]. Based on these methods, we introduced a modified radiographic measurement, periarticular soft tissue thickness ratio (PSTTR), to evaluate the soft tissue envelope size of the knee. Since the anatomical landmarks of patella were invalid in these patients, we evaluated soft tissue thickness of both femoral and tibial sides. The ratio was used in order to control for magnification variance. Femoral PSTTR (fPSTTR) and tibial PSTTR (tPSTTR) were measured separately on the lateral knee radiographs. The femoral side was measured by drawing a reference line perpendicular to the anterior cortex of femur from the most proximal portion of the posterior condyles. The width of the anterior soft tissue in line with the reference line was then divided by the width of the bone to yield the fPSTTR (Fig. 1: fPSTTR is BC divided by AB). The tibial side was measured by drawing a reference line in line with the tibial plateau. tPSTTR was defined as the ratio between the width of the anterior soft tissue in line with the reference line and the width of the bone (Fig. 1: tPSTTR is EF divided by DE). All radiographs were measured independently by two observers in a blinded fashion, and the average value was used in the analysis. Both of the observers were orthopedic surgeons, who were trained by the same researcher about the methods to measure fPSTTR and tPSTTR.

Statistical analysis was performed using Stata, version 13.0 (Stata Corp LP, College Station, TX). Gender, age, BMI, hypertension, diabetes, smoking status, AO/OTA classification, time to operation, preoperative level of CRP, time to X-ray test, surgeons, fPSTTR and tPSTTR were introduced into the univariate analysis to screen for risk factors associated with postoperative wound complication. Quantitative variables were compared using the t test, and qualitative variables were compared using the chi-square test. A receiver-operating characteristic (ROC) curve analysis was performed to evaluate the predictive value of PSTTR. Inter-observer and intra-observer reliability were examined using Pearson’s coefficients, and internal consistency was evaluated using a Cronbach’s alpha coefficients.

## Results

A total of 237 patients were included in this study, with 120 females and 117 males. The mean age was 53.1 ± 13.2 years (range: 12–85 years). The mean BMI was 24.4 ± 3.1 kg/m^2^ (range: 16.1–35.1 kg/m^2^). One hundred and forty-threefractures (60.3%) were classified as AO/OTA 34-C1, sixty-nine (29.1%) were classified as AO/OTA 34-C2, and twenty-five (10.5%) were classified as AO/OTA 34-C3. Fifty-three patients underwent removal of hardware for reasons other than infection. The average time from primary fracture fixation to removal of hardware in these patients was 15.7 ± 3.0 months (range: 12–24 months). The mean follow-up in other patients was 28.9 ± 13.9 months (range: 12–56 months). The demographic and clinical characteristics were summarized in Table 1.

**Table 1:** Patient’s demographic and clinical characteristics

Characteristics	Total (*n* = 237)
Gender	
Male (%)	120 (50.6)
Female (%)	117 (49.4)
Age, years (mean ± SD)	53.1 ± 13.2
BMI, kg/m^2^ (mean ± SD)	24.4 ± 3.1
Hypertension (%)	56 (23.6)
Diabetes (%)	34 (14.3)
Smoking history (%)	32 (13.5)
AO/OTA classification	
34C-1 (%)	143 (60.3)
34C-2 (%)	69 (29.1)
34C-3 (%)	25 (10.5)
Time to X-ray test, days (mean ± SD)	1.8 ± 1.6
Time to operation, days (mean ± SD)	5.9 ± 2.8
Preoperative level of CRP, mg/L (mean ± SD)	8.9 ± 13.1
Surgeon	
Surgeon 1 (%)	70 (29.5)
Surgeon 2 (%)	49 (20.7)
Surgeon 3 (%)	66 (27.8)
Surgeon 4 (%)	52 (21.9)
fPSTTR (mean ± SD)	0.94 ± 0.17
tPSTTR (mean ± SD)	0.66 ± 0.16

*n* Number, *SD*,Standard deviation, *BMI* Body mass index, *CRP* C-reactive protein, *PSTTR* Periarticular soft tissue thickness ratio, *fPSTTR* Femoral PSTTR, *tPSTTR* Tibial PSTTR

Twenty-four patients (10.1%) developed minor wound complication after ORIF of patella fracture. All of them received prolonged intravenous antibiotics, and bacterial cultures of the drainage were performed for each patient. Among these patients, bacterial cultures were negative in 20 patients. Staphylococcus aureus was cultured in the drainage of three patients and Enterobacter cloacae was cultured in the drainage of one patient. All the minor wound complications were controlled well by using intravenous antibiotics and intensive wound care. Only one patient (0.4%) received reoperation to remove the hardware for the major wound complication. The result of bacterial culture was Enterobacter cloacae in this case.

The mean fPSTTR was 0.94 ± 0.17 (range: 0.56–1.33) and the mean tPSTTR was 0.66 ± 0.16 (range: 0.29–1.09). Pearson’s coefficients for inter-observer were 0.967 for fPSTTR and 0.982 for tPSTTR. The intra-observer Pearson’s coefficients were 0.946 for fPSTTR and 0.977 for tPSTTR. Cronbach alpha scores were 0.983 for fPSTTR and 0.991 for tPSTTR, which indicated an excellent internal consistency. fPSTTR (*R* = 0.162) and tPSTTR (*R* = 0.048) showed a weak correlation with BMI.

**Fig. 2:**
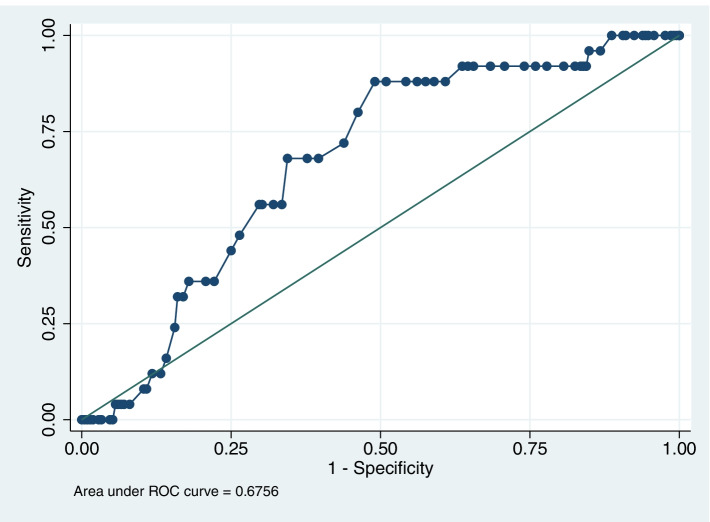
The ROC analysis of fPSTTR predicting postoperative wound complication

In the univariate analysis (Table 2), there was no significant association between BMI and wound complication (*P* = 0.676). The level of preoperative CRP (*P* < 0.001) and fPSTTR (*P* = 0.012) were found to be associated with wound complication. However, tPSTTR showed no statistically significant association with wound complication (*P* = 0.465). In the ROC analysis of fPSTTR predicting postoperative wound complication (Fig. 2), the area under curve (AUC) was 0.676, which indicated a moderate predictive value. When the cut-off value of fPSTTR was set at 0.94, the sensitivity and specificity were 88.0% and 50.9%, respectively.

**Table 2:** Univariate analysis of the risk factors for postoperative wound complication

Variable	Wound complication (*n* = 25)	No wound complication (*n* = 212)	*P* value
Male gender (%)	16 (64.0)	101 (47.6)	0.122
Age (mean years ± SD)	50.4 ± 11.2	53.4 ± 13.4	0.275
BMI, kg/m^2^ (mean ± SD)	24.7 ± 2.0	24.4 ± 3.2	0.676
Hypertension (%)	5 (20.0)	51 (24.1)	0.652
Diabetes (%)	4 (16.0)	30 (14.2)	0.803
Smoking history (%)	3 (12.0)	29 (13.7)	0.816
AO/OTA classification			0.177
34C-1 (%)	16 (64.0)	127 (59.9)	
34C-2 (%)	9 (36.0)	60 (28.3)	
34C-3 (%)	0 (0)	25 (11.8)	
Time to X-ray test, days (mean ± SD)	2.2 ± 1.3	1.7 ± 1.6	0.218
Time to operation, days (mean ± SD)	6.9 ± 3.8	5.8 ± 2.7	0.064
Preoperative level of CRP, mg/L (mean ± SD)	17.2 ± 20.3	7.9 ± 11.7	< 0.001*
Surgeon			0.793
Surgeon 1 (%)	7 (28.0)	63 (29.7)	
Surgeon 2 (%)	4 (16.0)	45 (21.2)	
Surgeon 3 (%)	9 (36.0)	57 (26.9)	
Surgeon 4 (%)	5 (20.0)	47 (22.2)	
fPSTTR (mean ± SD)	1.02 ± 0.12	0.93 ± 0.17	0.012*
tPSTTR (mean ± SD)	0.68 ± 0.14	0.66 ± 0.16	0.465

^*^P < 0.05 was considered statistically significant

*n* Number, *SD* Standard deviation, *BMI* Body mass index, *CRP* C-reactive protein, *PSTTR* Periarticular soft tissue thickness ratio, *fPSTTR* Femoral PSTTR, *tPSTTR* Tibial PSTTR

In the ROC analysis of fPSTTR predicting postoperative wound complication, the AUC was 0.676. When the cut-off value of fPSTTR was set at 0.94, the sensitivity and specificity were 88.0% and 50.9%, respectively.

## Discussion

In order to determine the association of periarticular adiposity and swelling with wound complications following ORIF of patella fractures, we established PSTTR to evaluate soft tissue status separately in the femoral and tibial side on lateral knee X-ray radiographs. According to the results, the increased fPSTTR was associated with postoperative wound complication. In the ROC analysis of fPSTTR predicting wound complication, the area under curve (AUC) was 0.676, which indicated a moderate predictive value.

The association between obesity and deficient wound healing has been well established. However, the patterns of body fat distribution vary from person to person, such as central or peripheral obesity. The mechanisms responsible for the obesity-related wound complications mainly focus on the soft tissue conditions at the surgical site [20]. Adipose tissue is characterized by its tenuous anatomic property and vascular insufficiency. The structure of fat lobules makes it susceptible to mechanical damage, which may cause adipose necrosis and subsequent inflammation [20]. The decreased vascularity of adipose tissue is the leading cause of delayed wound healing. Suppressed angiogenesis and microvascular abnormalities in adipose tissue may impair capillary recruitment after injury [20]. Besides, the poor vascularity of adipose tissue decreases oxygen tension, which impairs the synthesis of mature collagen and the production of immune mediators [21–23]. All of these factors may lengthen the inflammatory stage of wound healing and make injured site susceptible to infection. Therefore, locally excessive adiposity, instead of general obesity, may be responsible for wound complication. Even though BMI is the most commonly used tool to determine the presence and extent of obesity, it cannot reflect the local adiposity. Therefore, BMI may be insufficient to predict postoperative wound complication, which is also supported by this study. A more accurate tool to evaluate site-specific fat distribution is required when predicting wound complications.

The thickness of subcutaneous fat has proved to be associated with surgical site infection in patients undergoing colorectal surgery and acute appendicitis surgery [24–26]. Thicker subcutaneous fat thickness is also an independent risk factor for wound complications after lumbar spine surgery [27], and it could serve as a better indicator than BMI of the risk of surgical site infection [28]. Since BMI cannot reflect site-specific fat distribution, several studies have established different radiographic methods to evaluate subcutaneous fat thickness around knee in patients receiving total knee arthroplasty [17–19]. They found that the prepatellar and pretubercular soft tissue thickness correlated with postoperative wound complications in these patients.

In this study, we established a radiographic measurement to evaluate the periarticular soft tissue thickness. The ratios between soft tissue and bone were calculated separately in the femoral and tibial side. In this study, only fPSTTR was found to be associated with postoperative wound complication. The fPSTTR represents not only local adiposity but also hemarthrosis and soft tissue swelling. For the lower extremity fractures, soft tissue swelling has also been considered as the risk factor for wound complication [29]. Operation performed through swollen soft tissue may increase the risk of postoperative infection [30]. Therefore, the operation should be accomplished either before or after the period of excessive soft tissue swelling [31]. However, there is lack of quantitative indicators to evaluate the degree of soft tissue swelling. PSTTR may also serve as such an indicator.

There are several limitations in this study. First, this study has all of the limitations inherent to retrospective single-center study. Second, it is largely subjective to diagnose minor wound complication in clinical practice, which was much more common than major wound complication in this study. The treatment for wound complications was also based on the experience of surgeons. Therefore, different surgeons may chose different treatment strategies. Besides, patients in this study were followed up by routine clinic visit or telephone contact to collect information about wound complications. There may be missed event of wound complication, which leads to an underestimate of wound complication rate. Moreover, the application of PSTTR requires further validation in a larger population. In a large scale study, a multivariate analysis should be performed to control confounding effects of other factors. The potential role of PSTTR as a predictor for wound complication or other functional outcomes can also be explored in other types of periarticular fractures. In this study, we only included patients with standard lateral knee X-ray radiographs, which was taken when posterior femoral condyles overlapped and the knee was flexed between 10° and 30°. Further study is warranted to assess the impact of different knee flexion angles on the results.

## Conclusions

In conclusion, fPSTTR could be readily measured on the lateral knee X-ray radiograph and served as a quantitative indicator to evaluate soft tissue thickness around the knee. The increased fPSTTR was associated with wound complications after ORIF of patella fractures.

## Data Availability

The datasets used and/or analysed during the current study are available from the corresponding author on reasonable request.

## Abbreviations

ORIF: Open reduction and internal fixation
PSTTR: Periarticular soft tissue thickness ratio
fPSTTR: Femoral PSTTR
tPSTTR: Tibial PSTTR
BMI: Body mass index
IRB: Institutional Review Board
OTA: Orthopaedic Trauma Association
CRP: C-reactive protein
ROC: Receiver-operating characteristic
AUC: Area under curve
